# Neurologic and Psychiatric Manifestations of Bradykinin-Mediated Angioedema: Old and New Challenges

**DOI:** 10.3390/ijms241512184

**Published:** 2023-07-29

**Authors:** Ilaria Mormile, Francesco Palestra, Angelica Petraroli, Stefania Loffredo, Francesca Wanda Rossi, Giuseppe Spadaro, Amato de Paulis, Maria Bova

**Affiliations:** 1 Department of Translational Medical Sciences, University of Naples Federico II, Via S. Pansini 5, 80131 Naples, Italy; ilariamormile87@gmail.com (I.M.); f.palestra97@gmail.com (F.P.); ambulatoriopetraroli@gmail.com (A.P.); stefanialoffredo@hotmail.com (S.L.); francescawrossi@gmail.com (F.W.R.); spadaro@unina.it (G.S.); bovamaria@virgilio.it (M.B.); 2 Center for Basic and Clinical Immunology Research (CISI), WAO Center of Excellence, University of Naples Federico II, Via S. Pansini 5, 80131 Naples, Italy; 3 Institute of Experimental Endocrinology and Oncology “G. Salvatore” (IEOS), National Research Council (CNR), Via S. Pansini 5, 80131 Naples, Italy; 4 UOC Medicina 2, A.O.R.N. “Antonio Cardarelli”, Via Antonio Cardarelli, 9, 80131 Naples, Italy

**Keywords:** alteplase, autonomic disfunction, bradykinin, central nervous system, hereditary angioedema, psychology, psychiatry

## Abstract

Neurologic manifestations have been occasionally described in patients with bradykinin-mediated angioedema. The existing literature is currently limited to case series and case reports mainly described in the hereditary forms (HAE) concerning central nervous system (CNS) involvement. On the contrary, very little is known about peripheral and autonomic nervous system manifestations. CNS involvement in HAE may present with symptoms including severe headaches, visual disturbance, seizures, and various focal and generalized deficits. In addition, a stroke-like clinical picture may present in HAE patients. In turn, some drugs used in patients with cardiovascular and neurologic disorders, such as recombinant tissue plasminogen activator (r-tPA) and angiotensin-converting enzyme inhibitors (ACEI), may produce medication-induced angioedema, resulting in a diagnostic challenge. Finally, most patients with HAE have higher levels of psychological distress, anxiety, and depression. With this review, we aimed to provide an organized and detailed analysis of the existing literature on neurologic and psychiatric manifestations of HAE to shed light on these potentially invalidating symptoms and lay the foundation for further personalized diagnostic pathways for patients affected by this protean disease.

## 1. Introduction

Angioedema without wheals (AE) is caused by an increased vascular permeability due to the release of vasoactive mediators such as bradykinin [1]. This rare disease is clinically characterized by swelling of cutaneous and subcutaneous tissue involving several anatomical districts such as skin, gastrointestinal tract [2], respiratory airways [3], and urinary and genital tracts [4]. Bradykinin-mediated angioedema is classified into hereditary (HAE) and acquired forms (AAE) [1,5,6]. Hereditary forms are due to mutations in genes such as *SERPING1* (C1 esterase inhibitor; C1-INH-HAE), *F12* (factor XII; FXII-HAE), *PLG* (plasminogen; PLG-HAE), *ANGPT1* (angiopoietin 1; ANGPT1-HAE), *KNG1* (kininogen 1; KNG1-HAE), *MYOF* (Myoferlin; MYOF-HAE), and *HS3ST6* (heparan sulfate (HS)-glucosamine 3-O-sulfotransferase 6; HS3ST6-HAE) [7,8,9]. In other cases, the genetic background remains unknown (HAE-UNK). HAE attacks may be triggered by a wide variety of trigger factors such as emotional and physical trauma, surgical intervention, menstruation, drugs (i.e., angiotensin-converting enzyme inhibitors (ACEI) or estrogen-containing medications), and infections [10].

Bradykinin-mediated angioedema is unresponsive to conventional treatment administered in allergic angioedema, such as antihistamines [11,12]. Treatment strategies effective in bradykinin-mediated angioedema are mainly approved for the hereditary forms and include on-demand therapy for the treatment of acute attacks, short-term prophylaxis (STP) to prevent attacks during high-risk procedures such as surgical interventions, dental surgery, endotracheal intubation, bronchoscopy, or esophagogastroduodenoscopy; and long-term prophylaxis (LTP), in patients with frequent attacks in order to reduce the burden of disease [11,13]. Drugs for on-demand therapy include plasma-derived C1-inhibitor (pdC1-INH), icatibant, ecallantide, and recombinant human C1-INH (rhC1-INH) [11]. In countries where these first-line therapies are not available, other options are solvent-detergent-treated plasma (SDP) or fresh frozen plasma (FFP) [11]. STP may be performed using periprocedural pdC1-INH at the dose of 1000 units or 20 units/kg as the first line. Attenuated androgens (e.g., danazol) may be used as a second choice, while tranexamic acid is not recommended for both STP and LTP [11,14]. The first-line therapy for LTP is lanadelumab, an anti-plasma kallikrein monoclonal antibody [15,16]. Other strategies for LTP are subcutaneous or intravenous administration of pdC1-INH, generally twice-weekly [11,14], and berotralstat, an orally administrated plasma kallikrein inhibitor [17]. Androgen derivatives have been largely used for LTP, but they may be burdened by several side effects, especially when used for extended periods [11,18].

HAE patients show a broad clinical expression since the attacks may localize to numerous anatomical regions. Neurologic manifestations have been occasionally described, but the existing literature about this rare clinical presentation is limited to case series and case reports. In this review, we aim to analyze the complex relationship between neurological disease and bradykinin-mediated angioedema, including the uncommon neurologic presentation of HAE and the newly described AAE induced by fibrinolytic drugs.

## 2. Central and Peripheral Nervous Systems Involvement

### 2.1. Physiopathological Aspects

There are various forms of bradykinin-mediated angioedema. Among the hereditary forms, the best characterized is the one associated with the deficiency of C1-INH. C1-INH inhibits components of the fibrinolytic, clotting, and kinin pathways, which, when activated, cause an increase in blood bradykinin that, in turn, induces capillary leakage [19,20]. C1-INH belongs to the superfamily of serine protease inhibitors. Alterations in both proteases and their inhibitors are well-known in several human diseases, such as early-onset pulmonary emphysema, liver diseases, and HAE [21]. In addition, two human genetic diseases of the CNS have been related to mutations in components of the extracellular proteolytic system [21]. In recent years C1-INH has been postulated to exert some additional functions independent by protease inhibition, including an anti-inflammatory role through the enhancement of phagocytosis, the suppression of leukocytes chemotaxis, gram-negative sepsis and endotoxic shock, and the release of some inflammatory mediators such as tumor necrosis factor-alpha [22]. Serin proteases such as thrombin, plasminogen activators, and neuropsin play a role in the nervous system in both neural development stages and in adults, regulating neuronal survival and neural plasticity processes [23].

Bradykinin excites primary sensory neurons and induces the release of neuropeptides such as substance P, neurokinin A, and calcitonin gene-related peptide, which may contribute to the neurogenic inflammation linked to the migraine-like headache observed in some patients with HAE [24,25]. Moreover, bradykinin is a potent vasodilator peptide that acts through endothelial B_2_ receptors [19,20]. This vasodilation may further contribute to neurogenic inflammation through plasma protein extravasation, changes in endothelial cells, platelet aggregation, and the release of serotonin and other inflammatory neuropeptides and mediators [26].

Finally, the complement system also plays a role in the astrocyte cytotoxicity involved in the neuroinflammation and demyelination observed in neuromyelitis optica (NMO). Therefore, C1-INH is a promising target for developing novel therapies for this disease [27]. Indeed, inhibiting early steps in the classical complement pathway is particularly interesting because NMO pathogenesis involves NMO-IgG complement effector function, in which the antibody Fc region binds C1q to initiate complement activation [27].

**Figure 1:**
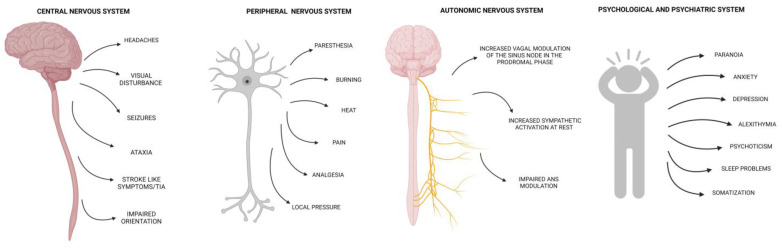
Neurological symptoms described in the literature up to now in hereditary angioedema patients. TIA—transient ischemic attack; ANS—autonomic nervous system.

Neurological involvement resulting from these molecular pathways may produce a broad clinical spectrum. Figure 1 summarizes the main neurological symptoms reported in the literature in patients affected by HAE.

### 2.2. Central Nervous System Involvement

#### 2.2.1. Central Nervous System Involvement in Hereditary Angioedema

Central nervous system (CNS) involvement in HAE has rarely been reported. Neurological symptoms commonly observed in HAE patients include severe headaches and visual disturbance [28], but seizures and a wide variety of focal and generalized deficits may occur [29].

Several case reports have associated stroke-like symptoms with HAE attacks. For instance, a case of local cerebral edema presenting with facial paralysis and hemiparesis was described in a female patient with HAE successfully treated with danazol 600 mg/day [30]. Previously, other similar cases have been reported [29]. Other insights came from the case of a female HAE patient with computed tomography finding suggestive of infarction in the right posterior cerebral artery described by Krause et al. [31]. Interestingly, her son, also diagnosed with HAE, showed episodes of prolonged reversible ischemic neurological deficit in the territory of the left middle cerebral artery causing transient ischemic attacks (TIAs). The rapid onset of local cerebral edema has been postulated to reduce blood flow in certain blood vessels, leading to TIAs [32]. In these cases, neurological symptoms are typically transient and resolve as the edema resolves. For example, Bonnaud et al. [32] reported a case of a patient with HAE presenting with repeated transient neurologic deficits, totally reverting after pdC1-INH administration. Although most of these authors observed the transitory nature of the neurological symptoms, a common feature of HAE attacks, some exceptions may occur. Lasek-Bal et al. [33] described the case of a C1-INH-HAE patient with dominant cerebral symptoms, finally leading to chronic disability secondary to focal brain ischemia and generalized cortical and subcortical atrophy.

The association between HAE and headache has been rarely investigated. Data from a large cohort of 221 patients with C1-INH-HAE analyzing a total of 121,110 angioedema attacks over a cumulative period of 5736 years showed that the frequency of headache episodes was 0.7% [34]. The accompanying symptoms described by the authors were feeling of pressure in the head or the eyes, visual disturbances (e.g., blurred vision, double vision, difficulty in focusing, and narrowed visual field), giddiness, vomiting, disorders of balance, ataxia, impaired orientation, and decrease in physical and mental powers. Despite the episodes being similar to migraine episodes, some clinical features typical of migraine were lacking. Indeed, no patient reported photophobia, sensitivity to noise, or an excessive urinary discharge following a headache episode [34]. The symptoms were unresponsive to analgesic and significatively improved after pdC1-INH administration [34]. This prompt response to pdC1-INH therapy suggests that the headache episodes may be an expression of HAE symptoms. In addition, the administration of danazol, which is used for the long-term prophylaxis of HAE, has been reported to prevent the recurrence of headaches [25].

HAE is a potentially life-threatening disease as laryngeal edema can cause death by asphyxiation, requiring specific and prompt therapy [35,36]. In addition, since bradykinin-mediated angioedema is unresponsive to conventional drugs used for treating histamine-induced angioedema (i.e., epinephrine, antihistamines), correct recognition of this condition is pivotal in the emergency department [37]. Although limited data are currently available in the literature, it is important to consider that HAE may also represent a neurological emergency causing a comatose state resembling a stroke [38]. In this case, an appropriate differential diagnosis can help initiate a specific treatment, such as C1-INH replacement therapy, with a complete resolution of the clinical picture.

#### 2.2.2. Iatrogenic Forms: Tissue Plasminogen Activator-Induced Angioedema

Thrombolysis is an effective therapy for eligible patients with ischemic stroke [39,40]. Recombinant tissue plasminogen activator (R-tPA), alteplase, and its genetically modified variant, tenecteplase, have been the main thrombolytic agents used by patients presenting with acute ischemic stroke who meet standard criteria for thrombolysis [41].

Fibrinolytic agents have been associated with a broad spectrum of allergic reactions [42]. In a large retrospective study by Duangmee et al. [42] analyzing 824 patients receiving fibrinolytic agents (i.e., streptokinase, alteplase, or tenecteplase) for various indications, streptokinase-caused type I hypersensitivity reactions in the 6.12% of cases. On the contrary, only one (0.18%) out of 547 patients treated with alteplase showed an adverse drug reaction consisting of angioedema in the face and lips in the absence of wheals [42]. Interestingly, the authors also reported 130 patients treated with tenecteplase, none of them presenting with type I hypersensitivity reactions and/or angioedema (i.e., in this group, only one patient (0.77%) developed hypotension as an adverse drug reaction) [42].

However, both alteplase [42,43,44,45,46,47] and tenecteplase [39] have been associated with the development of angioedema attacks without the concomitance of urticaria in various case reports and case series. In addition, some authors have reported that ACEIs, which are also responsible for a specific AAE form, may also increase the risk of oral angioedema attacks following the administration of tenecteplase or alteplase [43,45,46,47,48].

Angioedema is a condition that may potentially involve the airways. In hereditary forms, laryngeal attacks have been described in up to 35% of subjects [35]. In patients treated with thrombolysis for ischemic stroke, life-threatening episodes of swelling involving the tongue, lips, or the oropharynx have been described in 0.9–5.1% of cases [39,44,45]. A single report of gastrointestinal angioedema associated with the administration of alteplase for acute stroke has been described [49]. Although both alteplase and tenecteplase-related angioedema seem to involve the face [35,42,43,46], similarly to the more common ACEI-AAE, specific data regarding the different localization of the attack in this novel described AAE forms are scarce and need to be corroborated by further studies on large and homogeneous cohorts.

**Figure 2:**
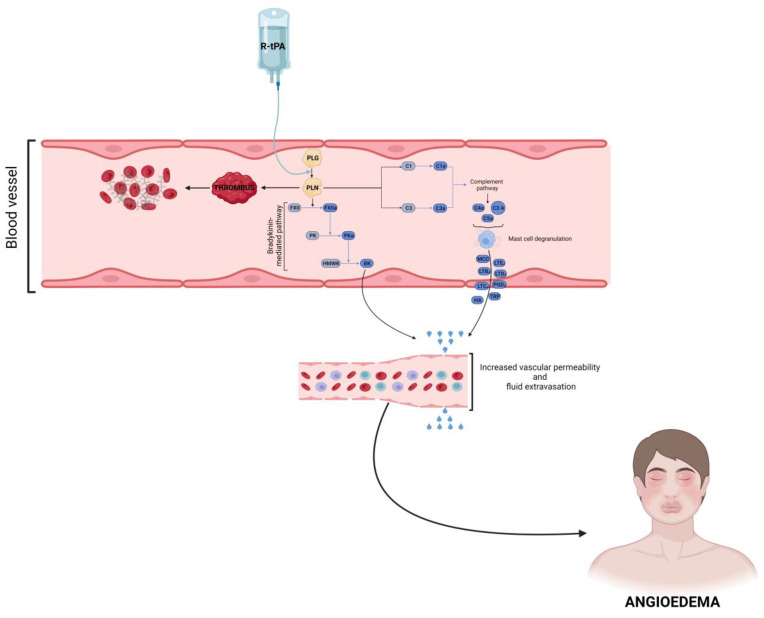
Proposed pathogenetic mechanisms of tissue plasminogen activator-induced angioedema. Recombinant tissue-type plasminogen activator (R-tPA) is a thrombolytic agent used to treat acute ischemic stroke. One of its side effects is the development of angioedema (AE). The figure shows the potential pathway involved in AE development. R-tPA converts plasminogen (PLG) to the proteolytic enzyme plasmin (PLN), resulting in fibrinolysis; PLG activation, in addition to fibrinolysis, may activate the kinin system, leading to bradykinin (BK) generation. PLN can also activate the complement system leading to mast cell degranulation with consequent release of histamine (HA), chymase (MCC), tryptase (TRP), cysteinyl leukotrienes (LTB4, LTC4, LTD4, LTE4), and prostaglandins (mainly prostaglandin D2 or PGD2). BK and mast cell degranulation mediators can increase vascular permeability and induce fluid extravasation, resulting in AE development. FDPs—fibrin degradation products; FXII—factor XII; FXIIa—factor XIIa; PK—prekallikrein; PKa—plasma kallikrein; HMWK—high-molecular-weight kininogen.

The underlying pathogenetic mechanisms of alteplase and tenecteplase-induced angioedema are unknown, but the activation of the complement pathway seems to be involved, together with increased production of bradykinin (Figure 2) [47,50].

According to the pathogenetic hypothesis, these molecules cleave plasminogen to plasmin, which induces the activation of factor XII to factor XIIa, which converts pre-kallikrein to kallikrein. In summary, R-tPA is responsible for increased plasma kallikrein levels which cause high-molecular-weight kininogen breakdown into bradykinin [49,51]. In addition, R-t-PA is also able to induce mast cell degranulation with consequent histamine release through the activation of the complement system and the release of anaphylatoxins. Indeed, fibrinolytic agents may also cause histamine-mediated reactions, including angioedema and anaphylaxis, which should always be considered in the differential diagnosis. An appropriate treatment choice in these different conditions could be complicated since antihistamines, corticosteroids, and epinephrine are considered ineffective in bradykinin-mediated angioedema [52]. Moreover, epinephrine itself may increase the risk of intracerebral hemorrhage and induce a sudden increase in blood pressure, making improper use of this drug seriously problematic. Data about severity and treatment approaches in R-tPA-induced angioedema are currently scarce. Some insights come from a retrospective study by Hurford et al. [47], including 530 consecutive patients receiving R-tPA for ischemic stroke. The authors reported that the attacks were mild in severity and that the administration of pdC1-INH and/or intramuscular epinephrine was required in a minority of cases. However, five patients presented with severe angioedema, requiring prompt endotracheal intubation or emergency cricothyroidotomy, and two died despite these interventions [47]. A case of alteplase-induced angioedema completely resolved following the administration of icatibant, a synthetic bradykinin B2-receptor antagonist effective in ACEI-AAE, given in association with adrenaline, has been described [53]. In conclusion, angioedema following thrombolysis often presents in a mild form, which could be self-resolving, but severe and life-threatening presentations are not uncommon [54]. For these reasons, increasing physicians’ awareness about this possible adverse event of fibrinolytic therapy is mandatory to ensure prompt recognition and appropriate management of this fearsome complication in an emergency setting.

### 2.3. Peripheral Nervous System Involvement

Very little is known about the peripheral nervous system involvement in patients with bradykinin-mediated angioedema. In HAE patients, neurosensory prodromes preceding the attacks of minutes or hours have been described in up to 31% of patients [55,56,57,58,59,60]. The symptoms reported were paresthesia, itching, burning, heat, pain, analgesia, and local pressure [61,62,63].

### 2.4. Autonomic Nervous System Dysfunction

Although it is well known that stressful events often trigger HAE attacks, the presence of an autonomic nervous system (ANS) dysfunction in these patients has rarely been investigated.

ANS exerts a role in the regulation of vascular permeability. In animal models, the sympathetic nervous system inhibition by clonidine, an alpha-2-agonist, reduces microvascular permeability stabilizing capillary leakage during inflammation. In addition, in models of inflammation such as ischemia–reperfusion injury, the vagus nerve has a protective role [64,65]. Moreover, bradykinin can modulate the parasympathetic tone by acting on B2 receptors in the ambiguous nucleus [66].

The possible relationship between ANS and the activation of the contact and complement system was first investigated by Wu et al. [67]. The authors continuously recorded ECG, beat-by-beat blood pressure, and respiratory activity during rest (10′) and 75°-head-up tilt (10′) in a cohort of 23 patients with HAE and assessed catecholamines in 16 of them. The results were compared with a control group. According to the results reported by the authors, HAE patients showed increased sympathetic activation at rest and blunted response to orthostatic challenge suggesting impaired ANS modulation [67]. Another interesting information provided by Wu et al. comes from the determination of C1-INH, C4, cleaved high molecular weight kininogen after the tilt test. Indeed, all the proteins tended to increase after the tilt test in both patients and controls, with a significant increase in cleaved kininogen only in patients suggesting a link between stress and bradykinin production [67]. All the evaluations conducted by Wu et al. were performed during the remission phase suggesting that autonomic dysfunction may exist as a constitutive characteristic of HAE patients. However, more recently, another research group assessed the cardiac autonomic profile during the attack or prodromal phases, performing a multi-day continuous electrocardiogram in four C1-INH-HAE patients until attack occurrence [65]. The authors reported an increased vagal modulation of the sinus node in the prodromal phase, possibly reflecting the localized vasodilation mediated by the release of bradykinin [65]. Finally, Frohlich and collaborators [39] used a voxel-wise lesion analysis to identify a major lesion cluster in the right insula associated with stroke-related angioedema, suggesting that an autonomic dysfunction due to insular infarction could have a role in the development of the swelling.

The relationship between ANS and HAE remains a grey zone, and further studies on larger cohorts are needed to explore if eventual alterations of ANS may contribute to the large variability of the rate and severity of the attacks observed in this condition.

## 3. Psychological and Psychiatric Manifestations

In recent years the interest in psychological aspects and implications on the quality of life of patients affected by chronic illness has substantially increased. On this path, many research groups have studied these aspects also in angioedema patients.

Savarese et al. [68] systematically reviewed the literature analyzing psychological aspects of C1-INH-HAE and their diagnostic and therapeutic implications. The studies treating these topics and included in the review showed that most patients affected by this condition have higher levels of psychological distress, anxiety, and depression than the normative sample [69,70,71,72]. Children with C1-INH-HAE have shown increased levels of stress compared with pediatric patients with other chronic diseases [73] and increased prevalence of alexithymia (i.e., the difficulties in recognizing and naming one’s own emotions) [74]; the latter also correlated with the severity of the disease. Interestingly, another study on an adult C1-INH-HAE patients group reported that alexithymia was absent in most patients (78%). In the same study, the psychopathology spectrum was also investigated using the SCL-90 R, a 90-item self-reported questionnaire, pointing out a high prevalence of psychoticism (48%) and low to moderate levels of sleep problems, somatization, paranoia, anxiety, and depression as a major area of symptomatology [75].

Emotional stress is a well-known trigger factor for HAE attacks [10,75,76]. The underlying molecular mechanisms behind this link are unknown, even though the increased production of bradykinin in stressful situations may be partially explained by the connection between ANS dysfunction and the activation of the contact/complement system discussed above [65,67]. The result is often a vicious circle between stress and the attacks leading to an increased disease severity due to the induction of more attacks. A survey by Banerji et al. [15] reported a direct correlation between the disease burden and attack frequency through administering the hospital anxiety and depression scale (HADS), showing that higher anxiety and depression scores were related to a higher attack frequency.

HAE is a potentially life-threatening disorder. In addition, many patients affected by the hereditary forms have ancestors who died of asphyxiation caused by laryngeal swelling [68]. For this reason, patients may live in constant fear of succumbing to a similar fate [77]. A C1-INH-HAE patients survey by Cicardi et al. [77] showed that the fear of airway obstruction and the dread of experiencing intolerable pain is one of the greatest concerns of these patients.

HAE is frequently misdiagnosed and confounded with diseases with a similar clinical presentation [78,79], the most common being allergic angioedema and appendicitis, as shown by data from the Icatibant Outcome Survey (IOS) study [79]. The same study also shows that patients with one or more misdiagnoses have significantly longer diagnostic delays than those without misdiagnoses, leading to otherwise avoidable economic, social, and psychological consequences [79]. In a study by Bygum et al. [80], misdiagnosis was reported by HAE patients to significantly increase disease burden, as it often implies unnecessary procedures, delays in appropriate treatments, and risk of death. HAE is indeed associated with a significant disease burden impairing quality of life, mental health, and decreased productivity, as also shown by a broad international survey recruiting 242 patients from Europe, Australia, and Canada [81].

Another common problem that aggravates the disease burden in some HAE cohorts is the loss of productivity [77,82,83]. Indeed, for the disfiguration and the often disabling symptoms suffered during the attacks, HAE patients may have some difficulties carrying out social activities and daily living [77,82,83]. Indeed, the unpredictable nature of the disease may make it difficult to manage the time between the attacks impacting patients’ choices in everyday life, such as avoiding traveling or starting some recreational activities [80].

Finally, in HAE patients, some psychiatric manifestations may be iatrogenic due to the administration of androgens (i.e., danazol), which have been largely used for long-term prophylaxis (LTP) [5]. Indeed, although most patients report an improvement in the severity and frequency of attacks with danazol, this drug is associated with several adverse effects, such as weight gain, virilization, menstrual irregularities, headache, depression, and/or liver adenomas [18]. Several patients may have psychological and behavioral abnormalities (e.g., aggressiveness) [18,84]. For this reason, it is recommended to plan a program for adverse effect surveillance when this drug is administrated. However, with the coming of new treatment options for LTP, the use of danazol has significantly dropped, as shown by the US physician survey, including 5382 physicians contacted during June and July 2019 (i.e., 55.8% in 2010, 23.4% in 2013; 6.4% in 2019, *p* < 0.001), resulting in less concern for adverse treatment effects and high levels of patient satisfaction [85].

## 4. Materials and Methods

This extensive literature review includes all articles from June 2023 and earlier, written in English, available in full-text, and published in peer-reviewed and international journals which dealt with neurologic and psychiatric manifestations of bradykinin-mediated angioedema. We searched for prospective and retrospective cohort studies, randomized control clinical trials, relevant systematic reviews, metanalysis, case reports, case series, and letters to the editor with a major focus or reported as secondary endpoints information about neurologic and/or psychiatric manifestations of bradykinin mediated angioedema and tissue plasminogen activator-induced angioedema. We searched three electronic bibliographic databases (PubMed, Scopus, and Web of Science). The search was conducted with the following combination of keywords: “hereditary angioedema”, “bradykinin-mediated angioedema”, “acquired angioedema”, “central nervous system”, “peripheral nervous system”, “autonomic dysfunction”, “psychology”, “psychiatry”, and “tissue plasminogen activator-induced angioedema”. We excluded studies dealing with histamine-mediated angioedema and type I hypersensitivity reactions. No restrictions based on publication date were applied. Two independent authors (IM and MB) selected candidate studies based on the inclusion and exclusion criteria mentioned above. Finally, the two main authors (IM and MB) agreed on the final selection of the studies.

## 5. Unmet Needs in Clinical Practice and Conclusions

Neurological symptoms of HAE represent a grey area that remains to be explored. The present data in the literature do not enable us to understand if they are extremely uncommon manifestations or underestimated expressions of this rare disease.

Planning a large multi-center randomized trial exploring the involvement of central, peripheric, and autonomic systems in patients with HAE is warranted for gaining insights into this little-covered topic and exploring the pathogenetic role of the complement system in developing some neurologic diseases.

On the contrary, the amount of literature about neuropsychiatric manifestations is wider, but many questions remain to be answered. HAE is a chronic, potentially life-threatening disease. Therefore, some alterations in the used score for anxiety and depression may be secondary to these aspects rather than specific features of HAE patients. In addition, HAE may present with large variability in clinical expression even in the same patient during different stages of life. This clinical heterogeneity, which is not always predictable, can create feelings of uncertainty, further increasing the burden of disease and acting as a confounder in assessing some psychological aspects of patients with HAE.

Another level of complexity is added since angioedema in patients with stroke may represent a diagnostic challenge. Indeed, some drugs used in patients with cardiovascular disease, such as R-tPA and ACE inhibitors, may produce medication-induced angioedema, and, in turn, a stroke-like clinical picture may present in patients with HAE. Some considerations further complicate the scenario. For example, it is important to consider that the mean diagnostic delay in HAE patients is 6.2–8 years [68,86,87]. Moreover, according to different cohorts, between 3% and 36% of patients are asymptomatic despite laboratory results indicative of HAE [4,88]. For these reasons, it could be assumed that these drugs sometimes unmasked an undiagnosed underlying condition rather than primarily inducing a de novo onset of angioedema. However, further large and homogeneous trials need to corroborate this hypothesis.

Last but not least, a better comprehension of the role of the complement in the pathogenesis of some neurologic disorders can pave the way for developing novel therapies for these diseases. An important role for the complement has been suggested in the pathogenesis of NMO, which has raised interest in the possible use of complement-targeted therapeutics, including eculizumab (a monoclonal antibody targeting complement protein C5) [89] and C1-INH [27]. NMO is an autoimmune disease of the CNS in which binding of anti-aquaporin-4 (AQP4) autoantibodies (NMO-IgG) to astrocytes causes complement-dependent cytotoxicity (CDC) and inflammation causing demyelinating lesions cause optic neuritis and transverse myelitis [90,91]. Most used treatments for NMO include glucocorticoids, human immunoglobulin, plasmapheresis, immunosuppressants (e.g., azathioprine, mycophenolate mofetil, methotrexate, cyclophosphamide), and monoclonal antibodies such as rituximab or tocilizumab [92]. pdC1-INH has been studied for acute NMO relapses in vitro and an experimental rat model (NCT 01759602), and then in a small safety trial involving ten patients administered 2000 units of pdC1-INH daily for three days, suggests a promising improvement with this drug in reducing neurologic damage and improving outcomes. [93].

With this review, we aimed to provide an organized and detailed analysis of the existing literature on neurologic and psychiatric manifestations of HAE to shed light on these potentially invalidating symptoms and lay the foundation for further personalized diagnostic pathways for patients affected by this protean disease.

## Data Availability

No new data were generated or analyzed for this review article.
